# Effect of Seed Priming with Chitosan Hydrolysate on Lettuce (*Lactuca sativa*) Growth Parameters

**DOI:** 10.3390/molecules28041915

**Published:** 2023-02-17

**Authors:** Tatiana Lyalina, Balzhima Shagdarova, Yuliya Zhuikova, Alla Il’ina, Alexey Lunkov, Valery Varlamov

**Affiliations:** Institute of Bioengineering, Research Center of Biotechnology of the Russian Academy of Sciences, 33, bld. 2 Leninsky Ave., 119071 Moscow, Russia

**Keywords:** chitosan, chitosan hydrolysate, polysaccharide, growth stimulant, seed priming, germination, lettuce (*Lactuca sativa*), chlorophyll, plant phenolics, enzyme activity

## Abstract

Seed priming increases germination, yield, and resistance to abiotic factors and phytopathogens. Chitosan is considered an ecofriendly growth stimulant and crop protection agent. Chitosan hydrolysate (CH) is an unfractionated product of hydrolysis of high-molecular-weight crab shell chitosan with a molecular weight of 1040 kDa and a degree of deacetylation of 85% with nitric acid. The average molecular weight of the main fraction in CH was 39 kDa. Lettuce seeds were soaked in 0.01–1 mg/mL CH for 6 h before sowing. The effects of CH on seed germination, plant morphology, and biochemical indicators at different growth stages were evaluated. Under the 0.1 mg/mL CH treatment, earlier seed germination was detected compared to the control. Increased root branching was observed, along with 100% and 67% increases in fresh weight (FW) at the 24th and 38th days after sowing (DAS), respectively. An increase in the shoot FW was found in CH-treated plants (33% and 4% at the 24th and 38th DAS, respectively). Significant increases in chlorophyll and carotenoid content compared to the control were observed at the 10th DAS. There were no significant differences in the activity of phenylalanine ammonia-lyase, polyphenol oxidase, β-1,3-glucanase, and chitinase at the 24th and 38th DAS. Seed priming with CH could increase the yield and uniformity of plants within the group. This effect is important for commercial vegetable production.

## 1. Introduction

Seed quality, abiotic stresses, and phytopathogens are factors influencing crop yields. Breeding and genetic engineering strategies for increasing crop yields and plant tolerance to biotic and abiotic factors are long-term and require a high level of technical equipment [1]. The cost of seeds is influenced by these factors. For this reason, simple, cost-effective, and quick techniques for treating seeds or seedlings are required to increase the productivity of field crops and tolerance to adverse environmental factors.

Pretreatment of seeds or seed priming can be used to increase the yield of crops in the field and in greenhouses by reducing the time of germination, as well as increasing seed germination and the uniformity of plant development [2]. An increase in plant resistance to phytopathogens can be achieved through priming. Priming affects the seed germination and seedling growth stages. These are important stages of crop development determining productivity. From an economic point of view, seed priming with growth regulators is profitable due to the lower consumption of formulation than foliar treatment. In addition, this technique is useful for substances with long preharvest intervals, which is especially important for crops with a short growing season, such as leafy vegetables.

Chitosan is a unique cationic polysaccharide of natural origin, composed of β-(1-4)-D-glucosamine (deacetylated unit) and N-acetyl-D-glucosamine (acetylated unit). Due to its biodegradability, biocompatibility, and low toxicity toward the environment, chitosan is a promising agrochemical. Chitosan enhances the immunity of plants and their resistance to abiotic stress, in addition to stimulating plant growth [3]. The bioactivity of chitosan depends on its structural parameters, including the molecular weight (MW), degree of deacetylation (DD), and the distribution of β-(1-4)-D-glucosamine and N-acetyl-D-glucosamine in the polymer chain. Chitosan acts as a plant growth stimulant for various crops including cereals, legumes, *Solanaceae*, and other crops, as described in [4,5,6,7,8,9,10]. The effect of chitosan depends on the plant species and treatment method. Biopolymers affect the growth rate, root development, and flowering, resulting in higher productivity of crops [11]. Chitosan induces the synthesis of secondary metabolites such as polyphenols, lignin, flavonoids, and phytoalexins. It directly affects gene expression by interacting with chromatin and/or binding to specific receptors [5].

In our work, leaf lettuce was chosen as the object of study. Lettuce is a source of vitamins, antioxidants, and dietary fiber [12]. Lettuce has a short period to horticulture maturity. This makes lettuce cost-effective crop for the provision of urban population needs of fresh vegetables. Priming of lettuce seeds with growth regulators can increase yield, synchronize and accelerate plant development, and increase the content of vitamins and phenolics.

The aim of the work was to study the effect of pretreatment of lettuce seeds with low-molecular-weight CH before sowing on the yield, morphological, and biochemical parameters of plants at different growth stages.

## 2. Results and Discussion

### 2.1. Seed Germination

**Figure 1:**
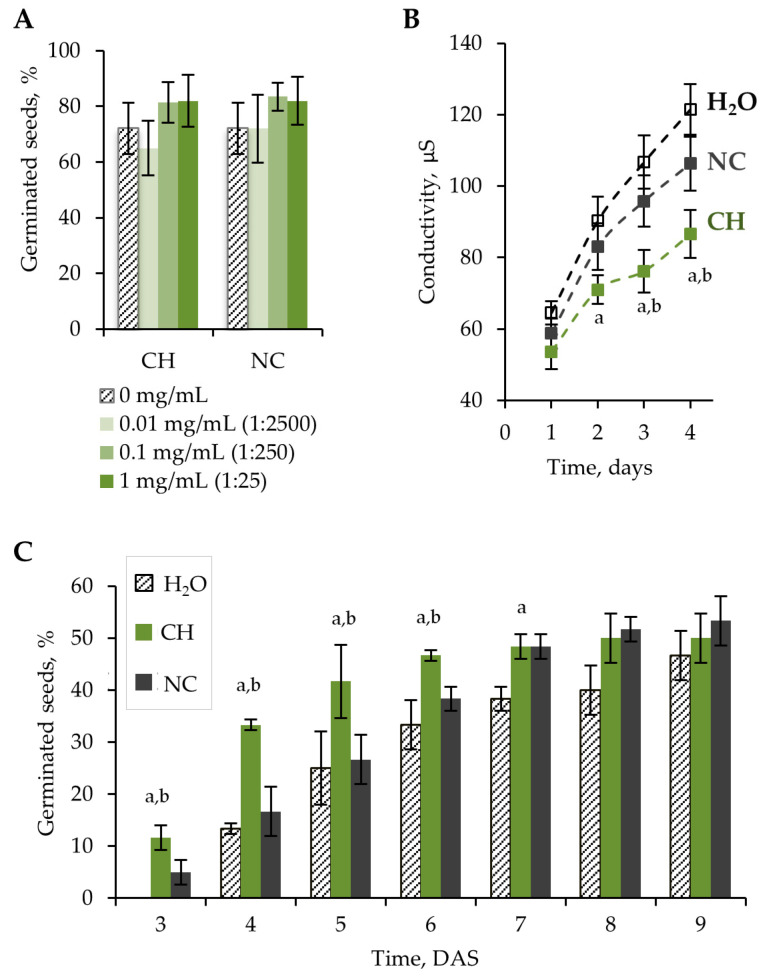
(**A**) Percentage of germinated seeds at the 10th day of incubation as a function of sample concentration in the germination test. (**B**) Conductivity of seed leachates at 1–4 days of incubation in distilled water. Seeds were treated with H_2_O, 0.1 mg/mL CH (dilution ratio 1:250), and NC (dilution ratio 1:250). (**C**) Percentage of germinated seeds at 3–9 DAS in peat substrate. Seeds were treated with H_2_O, 0.1 mg/mL CH (dilution ratio 1:250), and NC (dilution ratio 1:250). Error bars represent the standard deviation (SD) for three replications. Letters (a and b) indicate that values for CH-treated seeds are statistically significant compared to H_2_O- and NC-treated seeds, respectively, according to the Mann–Whitney U-test (*p* < 0.05).

The germination test was carried out according to the standard method described by the International Seed Testing Association [13]. Three different concentrations of chitosan hydrolysate (CH) were tested to reveal an effective concentration. Since CH also contains nitrogen in the form of ammonium nitrate, a nitrogen control (NC) was used. The NC was diluted to achieve a similar nitrogen concentration as in CH. Seeds soaked in distilled water or primed with water (H_2_O) were used as a control. Soaked lettuce seeds were germinated on wet filter paper. The germination of lettuce seeds was evaluated at the 10th DAS. There was no significant effect of CH on seed germination at concentrations of 0.01–1 mg/mL. An insignificant increase (*p* > 0.05) in germination was shown after CH and NC treatment at CH concentrations of 0.1 and 1 mg/mL (250- and 25-fold diluted solutions) (Figure 1A).

High chitosan concentrations cause suppression of seed germination. After treatments with chitosan (DD 98%) at fungicidal concentrations (5–20 g/L), an inhibitory effect on lettuce seeds compared to untreated seeds or those soaked with distilled water was shown [14]. This effect was observed due to the film-forming properties of concentrated chitosan solutions. The chitosan film prevents proper seed metabolism. 

The different pH values of the CH solutions probably did not affect seed germination, which correlates with earlier studies. Dias et al. [15] showed that, at pH values from 4.0 to 6.0, the acidity of the medium did not affect the overall germination of lettuce seeds, in contrast to osmotic pressure. In [16], it was shown that, except for extreme acidic pH values, there was no inhibition of germination. 

A slight increase in seed germination was observed at low chitosan concentrations according to the literature data. The efficacy of chitosan on seed germination was varied for the different crops. Unfortunately, in most cases, the authors did not show the characteristics of the used chitosan samples. Therefore, it is difficult to compare experimental data.

On lettuce seeds, a negative correlation between the germination percentage and the chitosan concentration or the contact time was observed in the article by Viacava et al. [17]. However, it was found that chitosan at a concentration of 0.01% allowed the seeds to maintain a high level of germination after 12–18 h of soaking, whereas, for water, germination decreased. A positive effect of chitosan on germination of common vetch (*Vicia sativa*) and lentil (*Lens culinaris* L.) was reported [18,19]. In these papers, a slight increase in seed germination was achieved by treatment with more concentrated chitosan solutions (1–8 mg/mL), which is probably due to the dense seed coat of legume crops. Cucumber seeds primed with chitosan at concentrations of 2.5 and 5 mg/mL showed significantly higher seed germination than control seeds treated with distilled water [20]. Fu et al. [21] showed that seed treatment with chitosan led to increased germination of wheat seeds. The authors also investigated the dependence of wheat seed germination on the DD of chitosan. It was shown that using low-molecular-weight chitosan with a DD of about 70% resulted in significantly higher germination compared to chitosan with a DD of 88%. For maize seeds, there was no significant difference in germination percentage irrespective of chitosan concentration (0–7.5 mg/mL) [6].

During the germination test, a higher radicle emergence rate was observed during the first 3 days after treatment with 0.1 mg/mL CH compared to H_2_O and NC (Figure S1). A significant increase (*p* < 0.05) in germination rate was noted at 3–6 DAS in peat substrate (Figure 1C).

Dehydration of seeds during development and storage, as well as rehydration of seeds during germination, causes changes in the organization of cell membranes [22,23]. Disruption of membrane integrity is one of the main causes of reduced seed viability. Changes in electrolyte leakage indirectly indicate membrane integrity. Lower electrolyte leakage indicates a higher rate of restoration of membrane integrity and, therefore, higher seed vigor [24,25]. The lowest electrolyte leakage was detected on seeds treated with 0.1 mg/mL CH (Figure 1B). This effect is probably related to a film formed on the seed surface that prevents ion exchange. Chitosan can also limit the swelling rate of seeds, thus reducing membrane damage during recovery after drying.

### 2.2. Influence of Chitosan on Plant Morphology

At the 10th day of cultivation on filter paper, morphological parameters such as root length and branching, hypocotyl length, embryonic leaf length, and the number of plants with true leaves were measured. The most significant differences in the root system development were observed (Table S1). At the CH concentration of 0.1 mg/mL, a significant increase (*p* < 0.05) in main root length was observed (56 mm for CH compared to 46 mm for H_2_O). At the same time, there was a minor increase (*p* > 0.05) in the number of plants with branched roots from 25% to 29%. CH caused an insignificant decrease (*p* > 0.05) in the size of embryonic leaves.

**Figure 2:**
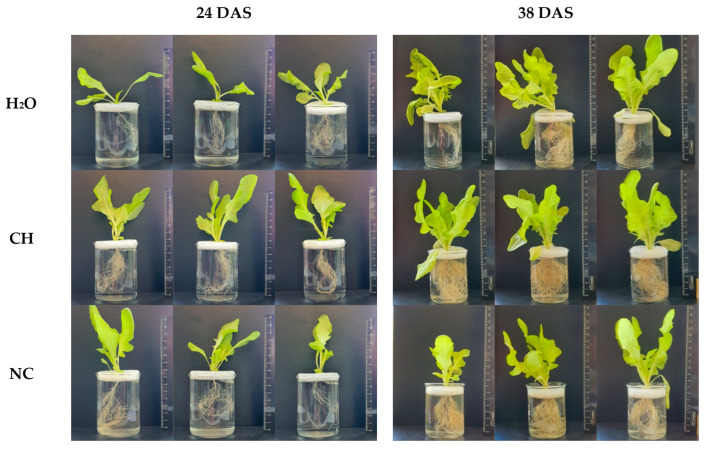
Influence of seed priming on development of shoots and roots of lettuce plants at the 24th and 38th DAS. CH concentration, 0.1 mg/mL (dilution ratio 1:250); dilution ratio of NC solution, 1:250.

Under the influence of CH, significant increases of 33% and 45% in the FW of above-ground plants relative to H_2_O and NC, respectively, were observed at the 24th DAS (Table 1). At the horticulture maturity stage at the 38th DAS, minor increases in FW by 4% relative to control and by 14% relative to NC occurred (Table 1). The shoot and root dry weight of CH-treated plants also increased at the 24th and 38th days (Table S2). Simultaneously, increases in shoot length, total leaf area, and the number of leaves in the rosette were determined. Along with the improvement of morphological parameters, there was also an increase in the uniformity of plants within the group (Figure 2). This effect is important for commercial vegetable production.

Seed priming with CH significantly enhanced root system development in plants at the 24th and 38th DAS (Table 1, Figure 2). The FW of root in CH-treated plants was twofold higher at the 24th DAS compared to plants treated with H_2_O and NC; at the 38th DAS, it was 67% and 29% higher compared to H_2_O and NC, respectively. At the same time, comparing the data on the length and weight of the root system, we can conclude that the presence of chitosan at the maturation stage had a greater influence on branching of the root system than on elongation of the roots.

In our opinion, there are several factors that can affect the development of the root system upon CH treatment. These factors can act individually or in a complex manner.

First of all, there is the direct effect of chitosan. The positive effect of chitosan with different characteristics on the length and branching of the root system, as well as on the root microstructure, has been previously described in a number of studies [26,27,28,29] on different crops. The authors in [30] found that the number of lateral roots, root length, root hair density, and total root weight of *Arabidopsis* and lettuce plants increased under the influence of chitosan (MW about 1500 kDa, DD > 87%) or chitosan microparticles at a concentration of 1–10 µg/mL. In contrast, in this paper, root development was observed to be impaired at concentrations above 10 µg/mL. This can be explained by the fact that the high-molecular-weight chitosan used by the authors had a greater ability to form a film on the seed surface, preventing normal metabolism. In our work, on the other hand, the CH preparation contained low-molecular-weight chitosan, which is less prone to the formation of polymer films. In contrast, Lopez-Moya et al. showed that medium-molecular-weight chitosan (MW 70 kDa and DD 85%) included in the nutrient medium inhibited the development of the root system of *Arabidopsis* and tomato [31]. In our opinion, this effect is due to the fact that chitosan can chelate metal ions from the nutrient medium or interact with other components of the medium, which can lead to plant nutritional deficiencies at a relatively high chitosan concentration.

Increased nitrogen level after CH or NC treatment is possibly the second factor influencing root development. Nitrogen, particularly nitrate ions, is known to be involved in the regulation of root branching at different stages by acting on auxin receptors [32,33].

Moreover, it was shown that, in lettuce seedlings, upon reducing pH from 5.5 to 4.0, the density and length of root hairs, as well as the number of lateral roots, increased, while the main root length was the same [34]. Considering this fact, we supposed that the relatively low pH of CH solutions in the used concentrations (4.8–5.2) may be another factor ensuring the active development of the root system.

### 2.3. Root Activity

Root activity (root energy) was assessed using thiazolyl blue tetrazolium bromide (MTT) reagent with respect to formazan formation (Table 2). At the 10th DAS, a dose-dependent increase in root activity of CH-treated plants was observed, but a significant difference (*p* < 0.05) from the control was observed only at a concentration of 1 mg/mL (0.36 OD units per g FW). Root activity varied from 0.28 to 0.36 OD units per g FW OD units per g FW for H_2_O and 1 mg/mL CH, respectively. Our findings are supported by other studies. An increase in root activity under the action of chitosan oligosaccharides was previously shown by Li et al. [35].

At the same time, plants treated with NC showed a higher level of root activity at 250- and 25-fold dilutions, reaching 0.46 OD units per g of FW. We can conclude that the increase in root activity in the early stages of development is associated with an increase in nitrogen levels and, to a lesser degree, with the action of chitosan.

Despite a significant increase in the root weight and the level of root system development under the action of CH, no differences in the level of root activity relative to the control were observed at the 24th and 38th days of cultivation. Herewith, a decrease in root activity during the period from 24th to 38th DAS was detected. We speculated that this was due to decrease in the cell-wall permeability to the dye. Reduced root activity in corn seedlings at later growth stages was also found [36].

### 2.4. Effect on Photosynthetic Pigments in Leaves

The effect of CH on photosynthetic pigments in leaves extracts was investigated at different stages of lettuce growth. At the 10th DAS, the content of total chlorophyll (Chl) and carotenoids (Car) in the shoots depending on the sample concentration was examined. An increase in the total Chl level from 283 to 371 μg/g FW was found when the concentration of CH was increased from 0.01 to 1 mg/mL, while, in the H_2_O-treated control, the total Chl content was 265 μg/g FW. The Chl content in plants from CH-treated seeds was higher compared to both H_2_O and to NC (Table 3). For the CH-treated plants, there was an increase in Chl *b* levels in all concentrations tested, regardless of the dose of the sample, at 105–110 μg/g FW, whereas, in the control, the average was 82 μg/g FW. There was a dose-dependent increase of Chl *a* content, under the action of CH to 242 μg/g FW at a CH concentration of 1 mg/mL. With increasing CH and NC concentrations, an increase in Car content relative to H_2_O-primed plants was observed, but the effect was lower for NC-treated plants. For CH, the Car level increased from 47 to 66 μg/g FW; for NC, it increased from 47 to 60 μg/g FW, while, in the H_2_O-primed plants, the Car content was 43 μg/g FW.

The increase in Chl content under the influence of CH pretreatment is in agreement with the data presented in the literature. Increases in total Chl, Chl *a*, and Chl *b* for sunflower plants at the 40th day at chitosan concentrations of 0.05–0.15 mg/mL were shown by Bakhoum [37], while they were shown for wheat plants at the 75th day at chitosan concentrations of 0.20 and 0.40 mg/mL [38]. 

Along with the increase in Chl content, there was a significant increase in the Chl *a/b* ratio in leaves of NC-treated plants from 2.1 in the H_2_O group to 2.3–2.4 in the NC group. There was also a decrease in the Chl/Car ratio for NC (Table 3). The Chl *a/b* ratio is often used to characterize the development state of the cell photosynthetic apparatus [39]. The Chl *a/b* ratio is fairly stable, but can vary depending on the physiological state of the plant; for example, it can decrease with leaf senescence [40].

The effect of an increased Chl *a/b* ratio was previously observed in a study of photosynthetic pigment content in leaves of plants at different light levels [41]. In this and other papers by these authors, it was shown that an increase in the Chl *a/b* ratio is associated with a change in the photosynthetic apparatus toward solar-type chloroplasts, which have fewer light-harvesting proteins. Leaves with solar-type chloroplasts had a significantly higher photosynthetic CO_2_ fixation rate. It can be assumed that CH is affected by the same mechanisms that lead to the formation of solar-type chloroplasts.

At the 24th day of cultivation, the highest level of photosynthetic pigments was detected (Table 4). At the same time, the values for CH were at the control level, while plants in the NC-treated group had the highest level of photosynthetic pigments. From the 24th to 38th days of cultivation, there was a reduction in total Chl and Car content, by 57% in H_2_O and NC, and by 37% in CH. The Chl *b* content at the 10th, 24th, and 38th days was at the same level for CH, whereas, for H_2_O and NC, there was an increase at the 24th day and a decrease at the 38th day.

We supposed that the decrease in photosynthetic pigment content was due to leaf senescence. Lettuce is a monocarpic plant whose final stage of development is seed formation. Leaf senescence is associated with the degradation of pigments, nucleic acids, proteins, and lipids. The degradation products of biomolecules are exported to the growing vegetative and reproductive organs of the plant as building material [42]. It was found that Chl *a* is generally more susceptible to degradation during senescence than Chl *b*, and that Car is relatively more stable than Chl [40].

Furthermore, the decrease in pigment content by the 38th day of cultivation (Table 4) can be related to an increase in light intensity, as the cultivation was carried out under combined light, under conditions of increasing intensity of natural light (April–May 2022). Previously, a decrease in Chl content in lettuce leaves under conditions of high light intensity was shown [43].

Another reason for the decrease in Chl level can be the depletion of nitrogen and other elements in the substrate during cultivation. Fontes et al. [44] showed a direct dependence of Chl content on the level of nitrogen in the substrate when the NH_4_NO_3_ content was increased from 60 to 960 mg N/kg soil. Becker et al. [45] showed that, during cultivation of lettuce at three different nitrogen levels of 12, 3, and 0.75 mM, Chl *a* and *b* content reduced with decreasing nitrogen concentration. The concentration of Chl *a* in plants grown at 0.75 mM was about 70% lower compared with 12 mM.

In general, it can be noted that, in the group of CH-treated plants, changes in pigment level over time were less prominent compared to the H_2_O and NC treatments. As a result, CH priming can not only increase biomass growth, but also maintain its properties for longer.

### 2.5. Effect on Protein Content in Leaves

The protein content in the lettuce leaves was determined using the Bradford method. The results of the study are presented in Table 5. At the 24th DAS, the plants treated with CH showed the highest level of protein. At the same time, the protein content in the NC-treated plants was 1.8 times lower than in the H_2_O-treated and two times lower than in the CH-treated plants. By the 38th DAS, the protein level in H_2_O- and CH-treated plants decreased by about 20% and 30%, respectively. For plants treated with NC, a nonsignificant (*p* > 0.05) increase in protein content was observed by the 38th DAS.

Our results are supported by other studies. An increase in protein content was observed after priming of mungbean seeds with chitosan compared to the control (no information was provided about the age of seedlings) [46]. However, the chitosan effect varied by cultivar. Chitosan treatment of pea seeds at concentrations from 0.05% to 1%, at the 38th day of cultivation, led to an increase in protein levels in the leaves [47]. 

The enzyme activity in the extracts was expressed per fresh leaf weight because of the significant differences in the protein content between the groups and the fact that the extract obtained from lettuce leaves is a mixture of proteins with different functions.

### 2.6. Phenolic Content and Activity of Enzymes of Phenolic Metabolism in Leaves

Phenolics are among the major classes of secondary metabolites with a high degree of structural heterogeneity. Phenolics in plants occur in many forms, ranging from simple phenolics to complex polymeric structures [48]. It is known that phenolics increase plant resistance to phytopathogens. They have antibacterial and fungicidal activity, induce the production of protective enzymes, and confer high resistance of cell walls to mechanical and enzymatic destruction. Phenolics are the basis of plant pigments and participate in the protection of plants from UV radiation, forming a kind of screen in plant epidermal cells. In addition, phenolics exhibit antioxidant properties, chelate metals, and are used by plants as signaling molecules, thus providing plant protection from abiotic factors [49]. The value of phenolics as a food component is determined by their effects on the organoleptic characteristics of fruits and vegetables, as well as functional properties such as antioxidant, anti-inflammatory, and antimicrobial [50].

In our study, the level of phenolics in lettuce leaves was determined at the 24th and 38th days of cultivation. At the 24th DAS, the level of phenolics in lettuce leaves was nearly equal between the groups, with a nonsignificant decrease (*p* > 0.05) for CH and NC. By the 38th DAS, an increase in total phenolics was detected (Table 5). At the same time, CH treatment resulted in more than a twofold increase in phenolics compared to the 24th DAS. At the 38th DAS, the content of total phenols was 1.5-fold higher in the CH group and 1.3-fold higher in the NC group compared to H_2_O-primed plants.

The change in level of phenolics varied depending on the chitosan concentrations. Treatment of pea seeds with chitosan at a concentration of 0.05% resulted in an increase in phenolic levels; however, at higher concentrations of chitosan (up to 1%), the phenolic levels decreased relative to controls as the chitosan concentration increased [47]. The chitosan effect on the total phenolics content in sweet basil plants at the 45th day of cultivation was shown. Total phenolic content increased significantly with chitosan treatment compared to water and 1% lactic acid. Total phenolics reached a maximum amount of 0.29 gallic acid equivalent (mg/mg fresh tissue) when treated with 0.1% chitosan. No further increase in total phenolic content was observed with increasing chitosan concentration of 0.5% or 1% [51].

It should be noted that the level of phenolics significantly depends on the stage of plant development. The increase in phenolic levels during maturation from the 24th to 38th day observed in our study is consistent with data from other authors [52,53,54,55]. The activity of phenylalanine ammonia-lyase (PAL), a key enzyme of phenylpropanoid pathway, which is directly involved in phenolic synthesis, was determined at the 24th and 38th days of cultivation at a CH concentration of 0.1 mg/mL (Table 5). On the 24th DAS, there was a reliable decrease in PAL activity in NC-treated plants. The decrease in PAL activity in CH group was insignificant relative to H_2_O. By the 38th day, PAL activity increased in all cases; however, for the NC group, PAL activity values remained lower than in H_2_O- and CH-primed plants. Thus, on the basis of the PAL activity in lettuce leaves at the maturation stage, we cannot conclude about the relation between the increase in phenolics and PAL activation under the action of chitosan.

In most works, the effect of chitosan on PAL was determined within a few hours or days after treatment. Numerous studies indicated an increase in PAL activity and upregulation of PAL gene expression under the action of chitosan in different crops and treatment techniques [20,56,57,58,59,60]. However, few studies examined the effects of chitosan over a longer period of time after treatment. In the study of Kim et al. [51], chitosan pretreatment was shown to increase PAL activity in sweet basil plants at the 45th DAS, at concentrations ranging from 0.1% to 1%, although PAL activity was similar to control values at lower chitosan concentrations of 0.01–0.05%. This is consistent with our results for 0.1 mg/mL CH. Thus, the difference between our data and those reported in the literature can be explained by the fact that the concentration of chitosan used in our work was much lower than that used in other works.

Phenolic compounds are substrates for polyphenol oxidase (PPO)-catalyzed oxidative reactions. In our study, the measured PPO activity in leaf extracts had no significant differences between the groups at both the 24th and the 38th days. At the same time, PPO activity increased in all groups by the 38th day compared with the 24th day (Table 5). The increase in PPO activity with maturation was shown earlier in [61] on kale (*Brassica oleracea* L. var. *acephala*), which confirms our data. An increase in PPO synthesis was detected with chitosan pretreatment (1–5 mg/mL) compared to control, but the enzyme activity was not measured by the authors [20].

It should be noted that PPO-catalyzed enzymatic browning reactions are important for agricultural producers because they reduce the attractiveness of products for consumers. Therefore, methods to prevention of browning are being developed. Chitosan treatment is considered as one of the possible methods against enzymatic browning reactions [62]. In our study, there was no increase in PPO activity under the action of CH compared to controls. Thus, CH had no effect on browning potential.

### 2.7. Effect on the Activity of β-1,3-Glucanases and Chitinases in Leaves

β-1,3-Glucanases and chitinases are important classes of hydrolytic enzymes. These enzymes belong to the so-called pathogenesis-related (PR) proteins that accumulate in various plant organs during infection with phytopathogens. Chitinases and β-1,3-glucanases play an important role in the defense response against the fungal pathogen by disruption of the fungal cell wall, whose major structural components are chitin and β-1,3-glucan [63].

β-1,3-Glucanase activity increased at the 24th day in the order H_2_O < CH < NC (Table 5). By the 38th DAS, β-1,3-glucanase activity in the H_2_O- and CH-treated groups was similar, while a slight decrease in activity relative to the water-treated control was observed in the NC group. At the 24th DAS, the chitinase activity tended to increase in the order H_2_O < CH < NC; an inverse dependence was observed at the 38th day of cultivation. The observed differences were not statistically significant at the 24th and 38th days, except for a decrease in chitinase activity in the NC group at the 38th day, which was significant (*p* < 0.05) relative to H_2_O.

Although the main interest in β-1,3-glucanases is related to their possible role in the plant response to microbial pathogens, these enzymes are also involved in various physiological and developmental processes in plants, including cell division, microsporogenesis, pollen germination, tube growth, fertilization, embryogenesis, fruit ripening, seed germination, mobilization of reserves in the endosperm of grain crops, resting buds, and response to injury, cold, ozone, and UV radiation [64,65].

In our study, the increase in chitinase and β-1,3-glucanase activities in the extracts of lettuce leaves by 38 days (Table 5) of cultivation was consistent with data from other studies. Thus, it was shown that β-1,3-glucanase and chitinase activities increase in tobacco with age [66]. Previously, it was shown that β-1,3-glucanase is a major component of protein in the lower leaves and roots, but not detected in leaves closer to the top of the plant [67]. Chun and Chandrasekaran [68] showed a significant increase in chitinase and β-1,3-glucanase gene expression in tomato whose seeds were pretreated with chitosan or chitosan-based nanoparticles after pathogen inoculation, compared to untreated plants exposed to the pathogen. 

In our research, no significant differences were revealed in enzyme activities among the CH-, NC-, and H_2_O-treated groups. Nevertheless, significant changes in plant growth parameters were shown. We suppose that the more evident changes in biochemical indicators occurred under the action of CH in early development stage immediately after sowing. However, this stage defines later changes in plant growth. In our future experiments, we plan to carry out a detailed study of chitosan’s action at the germination and early seedling growth stages.

## 3. Materials and Methods 

### 3.1. Chitosan Hydrolysate Preparation

High-molecular-weight crab shell chitosan (MW 1040 kDa, DD 85%) was obtained from Bioprogress (Shchelkovo, Russia). CH was prepared by depolymerization of high-molecular-weight chitosan with nitric acid as described previously [69]. Briefly, 10 g of chitosan was dispersed in 200 mL of 6.5% nitric acid, incubated for 7 h at 70 °C under stirring, cooled, and kept at room temperature for 16 h without stirring. The pH of the reaction mixture was adjusted to 5.2 with 25% ammonium hydroxide, before diluting with distilled water to a final volume of 400 mL. The final chitosan concentration in CH-stock solution was 25 mg/mL. The MW of chitosan in CH main fraction was 39 kDa, with a polydispersity index of 2.4 and DD of 90%.

For the determination of the MW and DD of chitosan in the main fraction of CH, CH was adjusted to pH 10 with 25% ammonium hydroxide for chitosan precipitation, after which the precipitate was dialyzed and freeze-dried. The MW of the CH main fraction was determined by high-performance gel permeation chromatography in a S 2100 Sykam chromatograph (Sykam, Eresing, Germany) using a separation column (8 mm × 300 mm; PSS NOVEMA Max analytical 1000 A) and a precolumn (8.0 mm × 50 mm) The 0.1 M NH_4_-acetate buffer + 0.2 M NaCl at 30 °C, pH = 4.5, was used for elution, with an elution rate of 1.0 mL/min. The analysis of chromatograms was carried out using the MultiChrom software version 1.6 (LLC Ampersand, Moscow, Russia). Pullulans (MW: 342, 1260, 6600, 9900, 23,000, 48,800, 113,000, 200,000, 348,000, and 805,000 Da) (PSS, Mainz, Germany) were used as calibration standards.

The DD was determined by the method of proton nuclear magnetic resonance (^1^H-NMR). The chitosan sample was prepared in deuterated water. The proton spectrum was recorded on a Bruker AMX 400 spectrometer (Bruker, Billerica, MA, USA); 4,4-dimethyl-4-silapentane-sulfonic acid was used as a standard. 

For biological studies, CH was diluted with distilled water to a concentration of 1 (pH 4.8), 0.1 (pH 4.9), and 0.01 (pH 5.2) mg/mL, obtained by diluting the CH stock solution 25, 250, and 2500 times, respectively.

Since CH contains inorganic nitrogen in the form of ammonium nitrate, we used a nitrogen control (NC) to eliminate the effect of inorganic nitrogen on the efficiency of CH. The NC solution was prepared by neutralization of nitric acid with ammonium hydroxide to pH 5.2. Briefly, 200 mL of 6.5% nitric acid was adjusted to pH 5.2 with 25% ammonium hydroxide, and the mixture was diluted with distilled water to a final volume of 400 mL. For analysis, the NC was diluted with distilled water 25 (pH 5.0), 250 (pH 5.25), and 2500 times (pH 5.4), in the same manner as the CH solution.

Distilled water was used as a control (H_2_O).

### 3.2. Germination Assay

The germination assay was carried out according to the germination test for lettuce, described in [13]. Immediately after seed soaking in CH, NC, and H_2_O, 50 lettuce seeds were sown in Petri dishes (diameter 8.5 cm) with two layers of filter paper, moistened with 3 mL of sterilized water. Then, the seeds were incubated in a germination chamber at 20–23 °C with a 16 h photoperiod for 10 days. The germination of seeds was determined by counting seedlings without defects at the 10th DAS of imbibed seeds. Morphological parameters of plants such as root length, presence of lateral roots, hypocotyl length, number of true leaves, and Chl content were determined at the 10th DAS.

### 3.3. Electrolyte Leakage from Lettuce Seeds

The effect of chitosan on electrolyte leakage from lettuce seeds was studied according to [22] with some modifications. Three groups of lettuce seeds (0.1 g) were soaked in the tested solutions (CH, NC, and H_2_O) for 6 h. The seeds were washed with distilled water and dried with filter paper. The seeds were placed in test tubes, and 50 mL of distilled water was added. The test tubes were incubated at 25 °C. The electrical conductivity of water was measured at 24, 48, 72, and 96 h after soaking using a conductivity meter Hanna HI 8733 (Hanna Instruments Inc., Woonsocket, RI, USA).

### 3.4. Plant Growth

Oak leaf lettuce (*Lactuca sativa* var. Dubachek MC) was grown under the experimental climate control facility in individual containers with 100 mL of universal peat substrate, at an air temperature of 20–23 °C, in natural light with additional lighting to maintain a 16 h photoperiod. Seeds were preliminarily soaked in the test solution or distilled water for 6 h, and excess moisture was removed from the seeds with filter paper. For the experiment, three groups of plants treated with CH, NC, and H_2_O were carried out. At the 24th and 38th DAS, four plants per group were sampled to determine total leaf area, shoot and root length, shoot and root FW, Chl and Car content, and enzyme activity. 

### 3.5. Leaf Area

Leaf area was estimated using a previously described method [70]. Each leaf was spread over graph paper, and the outline of the leaf was drawn. The area of the graph paper in the outline was cut and weighed on an analytical balance. A 1 cm^2^ piece of the same graph paper was also cut and weighed. Equation (1) was used to calculate the leaf area.

(1) $$\mathrm{Leaf}\;\mathrm{area}\;\left({\mathrm{cm}}^{2}\right)=\frac{x}{y},$$

where x is the weight of the graph paper cut in the outline (g), and y is the weight (g) of the 1 cm^2^ graph paper. 

### 3.6. Determination of Chlorophyll and Carotenoid Content in Lettuce Leaves

The amounts of Chl *a*, Chl *b*, and total Chl, as well as Car, were calculated in μg/g FW using the method described by Lichtenthaler [71]. For extraction, 0.1 g of lettuce leaves were homogenized in 2 mL of 95% ethanol. The suspension was centrifuged at 2000 rpm for 20 min. The contents of Chl *a*, Chl *b*, and total Chl were determined by spectrophotometry using the absorbance (Abs) at 664 nm and 648 nm according to Equations (2)–(4). Total Car was determined using the absorbance read at 470 nm and the data of Chl *a* and Chl *b*, as shown in Equation (4).

(2) $$\mathrm{Chl}\;a=13.36{\mathrm{Abs}}_{664}-5.19{\mathrm{Abs}}_{648}.$$

(3) $$\mathrm{Chl}\;b=27.43{\mathrm{Abs}}_{648}-8.12{\mathrm{Abs}}_{664}.$$

(4) $$\mathrm{Chl}=5.24{\mathrm{Abs}}_{664}-22.24{\mathrm{Abs}}_{648}.$$

(5) $$\mathrm{Car}=1000{\mathrm{Abs}}_{470}-2.13\mathrm{Chl}\;a-97.64\mathrm{Chl}\;b.$$

### 3.7. Determination of Total Phenolics in Lettuce Leaves

Total phenolics were measured using a previously described method [72] with some modifications. For preparation of the extraction solution, 2 mL of EtOH was mixed with 1 mL of 0.1 M HCl (final pH was 4.0). The extraction solution (0.2 mL) was added to 50 mg of plant material and kept in the freezer for 48–72 h. After freezing, each tissue sample was homogenized, and then 0.3 mL EtOH was added. Samples were centrifuged at 10,000 rpm for 15 min. One milliliter of supernatant from each of the centrifuged samples was assayed for total phenolic content. Then, 0.25 mL of EtOH and 1.25 mL of H_2_O were added to 0.25 mL of extract along with 0.125 mL of 1 N Folin–Ciocâlteu’s phenol reagent (Sigma Chemical Co., Burlington, Massachusetts, USA), vortexed, and allowed to incubate for 5 min. Next, 0.25 mL of 5% (*w*/*v*) sodium carbonate solution was added to each sample, vortexed, covered, and incubated in the dark for 1 h. The absorbance was read at 725 nm on a UV/Vis spectrophotometer (Shimadzu UV-1601PC spectrophotometer, Shimadzu Corp., Kyoto, Japan). A calibration curve was prepared with gallic acid (5–100 mg/mL), and the results were expressed as mg of gallic acid per g of FW.

### 3.8. Extraction of Biomolecules from Lettuce Leaves

Lettuce leaves (0.1 g) were homogenized in 750 μL of 0.05 M PBS (pH 7.4). Suspensions were centrifuged at 14,000 rpm for 10 min. Obtained extracts of lettuce leaves were used for analyses.

### 3.9. Determination of Protein Content in Lettuce Leaves

Protein content analysis was carried out according to the Bradford method [73] with minor changes. First, 600 µL of Coomassie G-250 dye in 3% HClO_4_ was added to 1400 µL of the diluted leaf extract. The mixture was left for 5 min before measurement. Optical density was measured at a wavelength of 595 nm (Leki SS1207UV spectrophotometer, Mediora OY, Tallinn, Estonia). The protein content was determined according to the calibration curve (BSA).

### 3.10. Polyphenol Oxidase Activity in Lettuce Leaves

The activity of PPO was measured using a previously described method [20] with some modifications. First, 66 μL of the leaf extract was added to 2 mL of 20 mM catechol solution in 0.025 M PBS (pH 6.8). The absorbance at 398 nm (l = 0.5 cm) was measured immediately (Shimadzu UV-1601PC spectrophotometer, Shimadzu Corp., Kyoto, Japan). The PPO activity was expressed as the increase in absorbance per sample FW.

### 3.11. β-1,3-Glucanase and Chitinase Activity in Lettuce Leaves

The measurements were carried out according to [74] with some modifications. To determine the chitinase activity, 30 µL of 0.1 M PBS at pH 6.0 and 30 µL of colloidal chitin (10 mg/mL) were added to 30 µL of the leaf extract. Samples were incubated for 60 min at 50 °C. To determine glucanase activity, 15 µL of 0.1 M Na-Ac buffer solution at pH 5.5 and 30 µL of 2 mg/mL laminarin solution were added to 15 µL of the extract. Samples were incubated for 45 min at 50 °C. Then, the reaction mixture was boiled for 10 min in a water bath to stop the reaction. The precipitate was separated by centrifugation at 5000 rpm for 10 min. The content of reducing sugars was determined by reaction with 3,5-dinitrosalicylic acid (DNS). For this purpose, 10 µL of the supernatant was mixed with 90 µL of water and 150 µL of DNS, before incubating in a boiling water bath for 20 min. After cooling, the content of reducing sugars was determined by absorbance at 540 nm on a Multiskan FC microplate photometer (Thermo Fisher Scientific Inc., Waltham, MA, USA). The calibration curve for N-acetylglucosamine was used to determine chitinase activity, and glucose was used as a standard for glucanase activity. One unit of enzyme was defined as the amount of enzyme required to release of 1 μg of reducing sugar equivalent in 1 min per 1 mg of raw tissue.

### 3.12. Determination of Phenylalanine Ammonia-Lyase Activity in Lettuce Leaves

The PAL activity was measured according to [20] with some modifications. Lettuce leaves (0.05 g) were homogenized in 375 μL of 0.05 M PBS (pH 7.4) containing polyvinylpyrrolidone (13 mg/mL) and 2-mercaptoethanol (5 mM). Suspensions were centrifuged at 14,000 rpm for 10 min. Then, 90 μL of borate buffer (0.1 M, pH 8.5) was added to 10 μL of the extract. Next, 0.9 mL of a 2 mg/mL solution of phenylalanine in the borate buffer was added to the diluted extract. The reaction mixture was incubated for 30 min at 37 °C. The reaction was stopped by adding 0.25 mL of 1N HCl. The optical density was measured at 290 nm. A solution of phenylalanine in the borate buffer with the addition of 0.25 mL of HCl was used as a reference solution. The content of cinnamic acid was evaluated according to the calibration curve.

### 3.13. Effect of Chitosan Treatment on Root Activity

Root activity was determined as described in [35,75] with some modifications. The MTT method was used to measure root activity. The middle parts of roots were cut into pieces about 1 cm in length, and 50 mg of roots were weighed per sample. Then, 1 mL of MTT (1 mg/mL in 0.1 M PBS, pH = 7.4) was added to the root samples. The samples were placed in the dark for 24 h. After incubation, samples were washed twice using distilled H_2_O. For formazan extraction, 1 mL of 95% ethanol was added, and then the samples were heated at 85 °C water bath for 10 min. Next, 20 μL of extract was diluted with 180 μL of EtOH. The light absorbance was measured at wavelength of 485 nm on a Multiskan FC microplate photometer (Thermo Fisher Scientific Inc., Waltham, Massachusetts, USA). The root vigor was calculated as an absorbance at 485 nm per sample FW.

### 3.14. Statistics

All experiments with plants were conducted at least in triplicate. The results were assessed using the methods of variation statistics with standard statistical programs, Microsoft Excel (Microsoft Corp., Redmond, WA, USA) and Statistica for Windows Version 6.0 (Stat Soft Inc., Round Rock, TX, USA). The mean value and standard deviation (SD) were determined. To assess the significance of differences between the samples, the Mann–Whitney U-test was used. Differences between the two compared values were considered statistically significant if the probability of their identity was less than 5% (*p* < 0.05).

## 4. Conclusions

In this work, the effect of chitosan hydrolysate on several growth parameters of lettuce plants after seed priming was analyzed. Seedling emergence was accelerated by CH. A strong influence of CH on root system formation was shown. There was an increase in root branching and an almost twofold increase in root fresh weight compared to water-treated plants. The investigation of the possible mechanism of chitosan action leading to changes in root development is a point of scientific interest. It would be useful to study the efficacy of CH on root vegetables due to its root-stimulating activity. A well-developed root system allows the plant to use the nutrients from the substrate more efficiently. The increase in leaf fresh weight in CH-treated lettuce plants correlated with an increase in root system development, and this was observed throughout the lettuce growth cycle. The strongest effect on the weight of the aboveground part of the plants was observed at the 24th day of cultivation. The FW of leaves of the CH-treated plants was 33% and 45% higher than that of the H_2_O- and NC-treated plants, respectively. Therefore, we assume that CH priming caused a reduction in the time needed to achieve horticultural maturity of lettuce. In addition, there was an increase in plant homogeneity within the group. Thus, CH led to an increase in lettuce yield; accordingly, CH is a promising ecofriendly growth stimulator for commercial vegetable production.

## Figures and Tables

**Table 1:** Effects of CH, NC, and H_2_O on shoot and root FW, shoot and root length, and total leaf area of the lettuce plants at the 24th and 38th DAS.

Parameter	24 Day			38 Day		
	H_2_O	CH *	NC **	H_2_O	CH *	NC **
Shoot length, mm	119 ± 15	140 ± 15 ^a^	124 ± 14	125 ± 10	131 ± 10	122 ± 19
Shoot FW, g	0.85 ± 0.29	1.13 ± 0.27 ^a^	0.78 ± 0.28	1.90 ± 0.60	1.97 ± 0.49	1.73 ± 0.69
Total leaf area, cm^2^	59 ± 13	83 ± 9 ^a^	60 ± 12	157 ± 41	161 ± 31	141 ± 52
Root length, mm	133 ± 27	144 ± 20	149 ± 41	127 ± 50	162 ± 23	156 ± 25
Root FW, g	0.075 ± 0.054	0.194 ± 0.002 ^a,b^	0.09 ± 0.01	1.047 ± 0.540	1.753 ± 0.051 ^a^	1.357 ± 0.426

^a^ Significant relative to H_2_O according to Mann–Whitney U-test (*p* < 0.05); ^b^ significant relative to NC solution according to Mann–Whitney U-test (*p* < 0.05); * CH concentration, 0.1 mg/mL (dilution ratio 1:250); ** dilution ratio of NC solution, 1:250.

**Table 2:** Effect of CH, NC, and H_2_O on root energy.

Group	Concentration, mg/mL or Dilution Ratio	10 DAS	24 DAS	38 DAS
H_2_O		0.28 ± 0.09	0.44 ± 0.08	0.22 ± 0.07
CH	1 (1:25)	0.36 ± 0.01 ^a^	-	-
	0.1 (1:250)	0.33 ± 0.04	0.43 ± 0.04	0.20 ± 0.02
	0.01 (1:2500)	0.30 ± 0.06	-	-
NC	1:25	0.46 ± 0.02 ^a^	-	-
	1:250	0.41 ± 0.01 ^a^	0.49 ± 0.07	0.20 ± 0.05
	1:2500	0.28 ± 0.08	-	-

^a^ Significant relative to H_2_O according to Mann–Whitney U-test (*p* < 0.05).

**Table 3:** Effect of CH, NC, and H_2_O on photosynthetic pigments in dependence of concentration at the 10th DAS.

Group	Concentration, mg/mL or Dilution Ratio	Total Chl, μg/g FW	Chl *a*, μg/g FW	Chl *b*, μg/g FW	Chl *a*/*b*	Car, μg/g FW	Chl/Car Ratio
H_2_O	-	265 ± 10	175 ± 20	82 ± 13	2.1 ± 0.1	43 ± 2	6.1 ± 0.1
CH	1 (1:25)	371 ± 16 ^a^	242 ± 30 ^a^	110 ± 14 ^a^	2.2 ± 0.1	66 ± 6 ^a^	5.3 ± 0.3
	0.1 (1:250)	345 ± 25 ^a^	228 ± 36 ^a^	107 ± 12	2.2 ± 0.1	56 ± 13 ^a^	5.9 ± 0.4
	0.01 (1:2500)	283 ± 45	190 ± 34	105 ± 25	2.0 ± 0.1	47 ± 15	5.3 ± 0.1
NC	1:25	321 ± 58	227 ± 38	94 ± 17	2.4 ± 0.1 ^a^	60 ± 11 ^a^	5.4 ± 0.4
	1:250	273 ± 65	179 ± 39	84 ± 18	2.3 ± 0.1 ^a^	50 ± 14	5.4 ± 0.1
	1:2500	265 ± 76	199 ± 33	85 ± 18	2.3 ± 0.1 ^a^	47 ± 17	5.5 ± 0.1

^a^ Significant relative to H_2_O according to Mann–Whitney U-test (*p* < 0.05).

**Table 4:** Effect of CH, NC, and H_2_O on photosynthetic pigments at different stages of cultivation.

Day	Group	Total Chl, μg/g FW	Chl a, μg/g FW	Chl b, μg/g FW	Chl a/b ratio	Car, μg/g FW	Chl/Car Ratio
10	H_2_O	265 ± 10	175 ± 20	82 ± 13	2.1 ± 0.1	43 ± 2	6.1 ± 0.1
	CH	345 ± 25 ^a^	228 ± 36 ^a^	107 ± 12 ^a^	2.2 ± 0.1	56 ± 13	5.9 ± 0.4
	NC	273 ± 65	179 ± 39	84 ± 18	2.3 ± 0.1	50 ± 14	5.3 ± 0.1
24	H_2_O	432 ± 38	299 ± 35	130 ± 12	2.3 ± 0.2	72 ± 9	6.1 ± 0.4
	CH	405 ± 37	275 ± 33	122 ± 18	2.3 ± 0.1	67 ± 13	6.0 ± 0.4
	NC	522 ± 30	369 ± 27 ^a^	161 ± 12 ^a^	2.3 ± 0.1	82 ± 6	6.3 ± 0.3
38	H_2_O l	186 ± 11	111 ± 10	75 ± 7	1.5 ± 0.3	22 ± 5	8.2 ± 1.9
	CH	255 ± 45	148 ± 27	103 ± 11	1.5 ± 0.3	30 ± 11	9.1 ± 2.2
	NC	222 ± 25	152 ± 21	91 ± 10	1.5 ± 0.2	25 ± 6	9.1 ± 1.7

^a^ Significant relative to H_2_O according to Mann–Whitney U-test (*p* < 0.05).

**Table 5:** Effect of CH, NC, and H_2_O on the biochemical parameters of leaves extract at the different stages of cultivation.

Parameter	24 Day			38 Day		
	H_2_O	CH	NC	H_2_O	CH	NC
Total protein, μg/g FW	839 ± 76	958 ± 89 ^a^	472 ± 48	679 ± 66	678 ± 136	554 ± 154
Total phenolics, mg/g FW	4.1 ± 1.3	3.2 ± 0.9	3.5 ± 1.4	5.7 ± 1.1	8.8 ± 1.4 ^a^	7.6 ± 1.1
PAL activity, μg cinnamic acid/g FW	38.8 ± 9.4	34.0 ± 6.9	24.3 ± 7.7	127.3 ± 9.9	124.7 ± 23.0	117.8 ± 15.3
PPO activity, OD units/g FW	0.19 ± 0.04	0.21 ± 0.04	0.25 ± 0.10	0.74 ± 0.09	0.75 ± 0.05	0.6 ± 0.12
β-1,3-glucanase activity, μg glucose/g FW/min	0.88 ± 0.08	1.08 ± 0.14	1.34 ± 0.09	1.68 ± 0.12	1.72 ± 0.19	1.57 ± 0.10
Chitinase activity, μg glucosamine/g FW/min	0.53 ± 0.19	0.66 ± 0.17	0.76 ± 0.13	1.42 ± 0.06	1.27 ± 0.11	1.16 ± 0.06 ^a^

^a^ Significant relative to H_2_O according to Mann–Whitney U-test (*p* < 0.05).

## Data Availability

Not applicable.

## Supplementary Materials

The following supporting information can be downloaded at https://www.mdpi.com/article/10.3390/molecules28041915/s1: Figure S1. Percentage of seeds with emerged radicle in the germination test. Seeds were treated with H_2_O, CH 0.1 mg/mL (dilution ratio 1:250), and NC (dilution ratio 1:250). Error bars represent the standard deviation (SD) for three replicates; Table S1. Effects of CH, NC, and H_2_O priming on the morphology of lettuce plants at 10 days after sowing. Seedlings were grown on moistened filter paper; Table S2. Effects of CH-, NC- and H_2_O -priming on the shoot and root dry weight of lettuce plants at the 24th and 38th days after sowing.

## Sample Availability

Samples of the compounds are available from the authors.
