# Supplementary material for: Effect of Seed Priming with Chitosan Hydrolysate on Lettuce (Lactuca sativa) Growth Parameters
Source: Molecules. 2023 Feb 17;28(4):1915. doi: 10.3390/molecules28041915 (PMC9959803; doi:10.3390/molecules28041915)
Supplement: Supplementary file 1 [file molecules-28-01915-s001.zip › molecules-2212855-supplementary.pdf]

molecules

Supplementary materials

Effect of Seed Priming with Chitosan Hydrolysate on Lettuce (*Lactuca sativa*) Growth Parameters

Tatiana Lyalina *, Balzhima Shagdarova, Yuliya Zhuikova, Alla Il'ina, Alexey Lunkov and Valery Varlamov *

Institute of Bioengineering, Research Center of Biotechnology of the Russian Academy of Sciences. 33, bld. 2 Leninsky Ave., Moscow 119071, Russia

* Correspondence: t.s.lyalina@gmail.com (T.L.); varlamov@biengi.ac.ru (V.V.); Tel.: +7 (499)135-6556

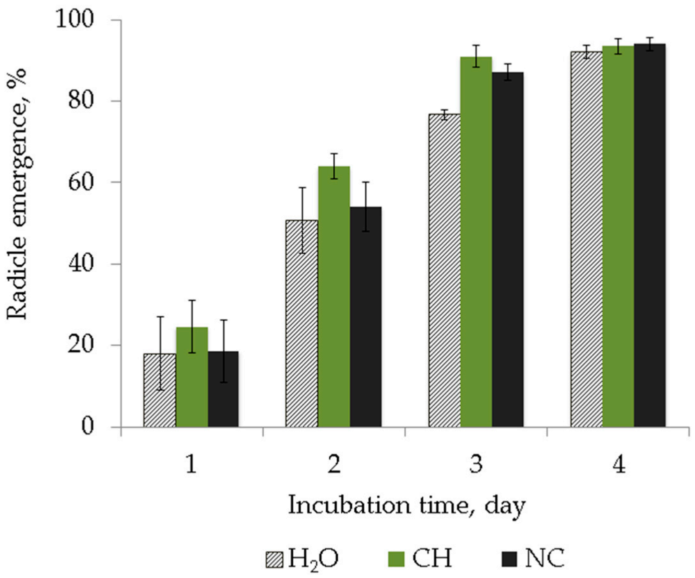

Figure S1. Percentage of seeds with emerged radicle in the germination test. Seeds were treated with H_2O, CH 0.1 mg/mL (dilution ratio 1:250) and NC (dilution ratio 1:250). Error bars represent the standard deviation (SD) for three replicates.

Table S1. Effects of CH-, NC- and H_2O -priming on the morphology of lettuce plants at 10 d after sowing. Seedlings were grown on moistened filter paper.

**Parameters**	**H_2O**	**CH concentration mg/ml (dilution ratio)**			**NC dilution ratio**		
		**0.01 (1:2,500)**	**0.1 (1:250)**	**1 (1:25)**	**1:2,500**	**1:250**	**1:25**
Root length, mm	46.2±6.7	48.7±8.4	56.4±2 [a]	54.8±7.5	52.4±7.1	53.7±3.8	47.6±2.9
Branched root plants,%	25.1±5.0	18.8±5.0	29.2±7.5	29.2±4.0	20.7±3.6	27.7±8.5	26.5±10.1
Hypocotyl length, mm	1.8±0.2	1.7±0.1	1.6±0.2	1.7±0.3	1.6±0.4	1.9±0.3	1.6±0.3
Cotyledon length, mm	5.7±0.7	5.1±0.6	5.5±0.6	5.3±0.3	5.4±0.8	5.6±0.5	5.0±0.7
Plants with true leaves, %	54.8±5.4	54.5±16.6	54.6±9.9	58.4±16.6	49.6±15.7	43.9±15.6	47.4±29.2

[a] Significant relative to H_2O according to Mann-Whitney U-test ($p < 0.05$).

Table S2. Effects of CH-, NC- and H_2O -priming on the shoot and root dry weight of lettuce plants at the 24th and 38th days after sowing.

**Parameter**	**24 day**			**38 day**		
	**H_2O**	**CH***	**NC****	**H_2O**	**CH***	**NC****
Shoot DW, mg	44±16	69±17[a]	43±7	283±23	404±30 [a]	359±133
Root DW, mg	7±5	13±0.2[a,b]	8±1	113±58	210±6[a]	171±54

[a] Significant relative to H_2O according to Mann-Whitney U-test ($p < 0.05$).

[b] Significant relative to NC solution according to Mann-Whitney U-test ($p < 0.05$);

* CH concentration – 0.1 mg/mL (dilution ratio 1:250);

** Dilution ratio of NC solution 1:250.
